# Racial difference in BMI and lung cancer diagnosis: analysis of the National Lung Screening Trial

**DOI:** 10.1186/s12885-022-09888-4

**Published:** 2022-07-19

**Authors:** Joy Zhao, Julie A. Barta, Russell McIntire, Christine Shusted, Charnita Zeigler-Johnson, Hee-Soon Juon

**Affiliations:** 1 Sidney Kimmel Medical College, Thomas Jefferson University, 1101 Locust Street, Philadelphia, PA USA; 2 Division of Pulmonary and Critical Care Medicine, Jane and Leonard Korman Respiratory Institute, Thomas Jefferson University, 834 Walnut Street, Philadelphia, PA USA; 3 Jefferson College of Population Health, Thomas Jefferson University, 901 Walnut Street, Philadelphia, PA USA; 4 Division of Population Science, Department of Medical Oncology, Thomas Jefferson University, 834 Chestnut Street, Philadelphia, PA USA

**Keywords:** BMI, Race, Lung cancer diagnosis, NLST

## Abstract

**Background:**

The inverse relationship between BMI and lung cancer diagnosis is well defined. However, few studies have examined the racial differences in these relationships. The purpose of this paper is to explore the relationships amongst race, BMI, and lung cancer diagnosis using the National Lung Screening Trial (NLST) data.

**Methods:**

Multivariate regression analysis was used to analyze the BMI, race, and lung cancer diagnosis relationships.

**Results:**

Among 53,452 participants in the NLST cohort, 3.9% were diagnosed with lung cancer, 43% were overweight, and 28% were obese. BMI was inversely related to lung cancer diagnosis among Whites: those overweight (aOR = .83, 95%CI = .75-.93), obese (aOR = .64, 95%CI = .56-.73) were less likely to develop lung cancer, compared to those with normal weight. These relationships were not found among African-Americans.

**Conclusion:**

Our findings indicate that the inverse relationship of BMI and lung cancer risk among Whites is consistent, whereas this relationship is not significant for African-Americans. In consideration of higher lung cancer incidence among African Americans, we need to explore other unknown mechanisms explaining this racial difference.

## Background

The prevalence of obesity, as defined by Body Mass Index (BMI) ≥ 30, among US adults in 2017–2018, was 42.4% [1]. Obesity is associated with increased risk of multiple cancers, including endometrial cancer [2], liver cancer [3], kidney cancer [4], multiple myeloma [5], pancreatic cancer [6], and colorectal cancer [7]. However, in lung cancer, which is the 2^nd^most frequently diagnosed cancer in both men and women [8], it has been well documented that there is an obesity paradox, or an inverse association between BMI and lung cancer risk [9–14]. More specifically, among current or former smokers, overweight or obese patients may have decreased risk of lung cancer [9, 10, 13].

Multiple studies have demonstrated that a greater BMI is significantly associated with lower risk of developing lung cancer [9, 11, 13, 15, 16]. A prospective cohort case–control study also demonstrated a decreased risk of lung cancer for overweight and obese patients among current, former, and never smokers [10]. The National Institutes of Health AARP Diet and Health Study, a prospective cohort study, likewise found that a BMI ≥ 35 kg/m^2^at baseline was inversely associated with lung cancer incidence for both men and women, and this effect was more substantial after adjusting for current vs. former smoking status [12].

To our knowledge, no studies have examined whether the obesity paradox exists in a lung cancer screening population. The National Lung Screening Trial (NLST) was a randomized, controlled trial comparing low-dose computed tomography (LDCT) with chest radiography in current and former heavy smokers [17]. Annual LDCT screening of high-risk individuals leads to a stage shift in lung cancer diagnosis and reduces lung cancer mortality [18, 19]. Moreover, the PLCOm2012 risk model includes BMI and found that a lower BMI was associated with an increased risk of lung cancer [20]. Therefore, identifying a potential obesity paradox in NLST data would be valuable as an identifiable lung cancer protective factor for screened patients.

Meta-analyses of previously published studies and a case–control study have stratified data based on smoking status and gender [9, 10, 13], but few studies have stratified by race. Only a single pooled analysis of twelve cohort studies examined this relationship and found a stronger obesity paradox in African-Americans than among White or Asian individuals [21]. Notably, African-Americans have a greater annual incidence of lung cancer compared to other races and ethnicities, with 76.1 per 100,000 people affected [22]. The objective of this study was to identify whether obesity was associated with screen-detected lung cancers among African-American and White participants in the NLST.

## Methods

### National Lung Screening Trial

The NLST study design has been described in detail previously [17]. Inclusion criteria were as follows: age 55 to 74 years and current or former smoker with at least a 30 pack-year history; former smokers had to have quit within the past 15 years. Screening, either LDCT or chest radiography, was offered to NLST participants annually for 3 consecutive years. The median follow-up time was 7 years. Approval for this project was obtained from the National Cancer Institute’s Cancer Data Access System on October 16, 2017 (NLST-361) and renewed on November 2, 2020.

### Measures

The NLST dataset provides a longitudinal perspective on high-risk lung cancer patients in terms of demographics, clinical history, and imaging data. Information used in our study includes demographic characteristics and risk factors for lung cancer development.

Outcomes. Lung cancers were identified as pulmonary nodules and confirmed by diagnostic procedures (e.g., biopsy, cytology); participants with confirmed lung cancer diagnoses were subsequently removed from the trial for treatment. Lung cancer diagnosis was defined as the number of cases determined to have cancer during any of the three imaging points of intervention (and the remaining number of non-cancer patients), as well as post-screening cancer patients (i.e., those individuals who went on to develop lung cancer after the third screening event).

BMI*. *The BMI groups were defined by the World Health Organization as follows: Underweight (BMI < 18.5), normal weight (BMI = 18.5–24.99), overweight (BMI = 25–29.99), and obese (BMI ≥ 30 +) [23]. In this analysis, we combined underweight with normal since less than 1% of NLST participants (*n* = 471) were underweight. We also excluded 326 participants who did not have BMI recorded.

Race. Race was constructed using two variables of race and ethnicity. These were 3 groups: non-Hispanic Whites, non-Hispanic African-Americans, and Others (e.g., Asian, Native Hawaiian or Pacific Islander, American Indian, Hispanic, or more than one race).

Control variables. Age, gender, smoking status, education, family history of lung cancer, pack-years of smoking, and having COPD were included as covariates. Age and pack-years of smoking were used as continuous variables.

### Statistical analysis

We used descriptive and analytic statistical methods in this study. Frequency and cross-tabulation were used to summarize descriptive statistics in tables. First, we examined whether BMI was associated with race and lung cancer development, including lung cancer stage and histological type. Then, we conducted multivariate logistic regression to estimate the effect of race and BMI on lung cancer diagnosis while controlling for potential confounders such as age, gender, family history of lung cancer, COPD, smoking status, and pack-years of smoking. Finally, we conducted subgroup analysis by race. We used Stata version 17 for statistical analyses.

## Results

The NLST baseline characteristics of participants have been previously described [17]. Of the total of 53,452 participants, mean age of the total cohort was 61.42 years (SD = 5.02 years), and 59% were men. 90% were non-Hispanic Whites, 4.4% were non-Hispanic African-Americans, and the remaining 5.6% were Others. Only 6% did not have a high school degree, and about 32% had at least a college degree. More than one-fifth had a family history of lung cancer. The mean smoking intensity was 55.9 pack-years, and about 5% had Chronic Obstructive Pulmonary Disease (COPD). Of the total cohort, 3.9% were diagnosed with lung cancer (Table 1).

**Table 1:** Background and clinical characteristics and lung cancer diagnosis in the National Lung Screening Trial (NLST) (*n* = 53,452)

	**n**	**%**
**Male Gender**	31,530	59.0%
**Age (mean ± SD)**	61.42 ± 5.02	
**Race/ethnicity**		
Non-Hispanic Whites	47,744	90.0%
Non-Hispanic African Americans	2,341	4.4%
Other Races	2,942	5.6%
**Education**		
< High school	3,249	6.2%
High school graduate +	32,423	62.1%
College graduates	16,546	31.7%
**Smoking status**		
Former	27,692	51.8%
Current	25,760	48.2%
**Smoking pack-years** (mean ± SD), range	55.9 ± 23.9	
**Family history of lung cancer** (= Yes)	11,037	20.7%
**COPD** (= Yes)	2,690	5.1%
**BMI (*n* = 53,090)**		
Underweight/Normal (< 25)	15,320	28.9%
Overweight (25–29.99)	22,761	42.9%
Obese (> = 30)	15,009	28.2%
**Lung cancer diagnosis**	2,058	3.9%
**Lung cancer stage**		
Stage I	831	40.4%
Stage II	146	7.1%
Stage III	459	22.3%
Stage IV	596	28.9%
No stage recorded	26	1.3%
**Histology**		
Adenocarcinoma	902	44.4%
Squamous cell carcinoma	462	22.7%
Large cell carcinoma	52	2.6%
Small cell carcinoma	287	14.1%
Carcinoid/ Neuroendocrine tumor	60	3.0%
Non-small cell carcinoma or other	271	13.3%

### BMI, race, and lung cancer development

Of the cohort of 53,090 participants, about 43% were overweight, and 28% were obese (Table 1). There was a significantly different distribution of BMI among racial groups, with 33.9% of African-Americans and 28.1% White individuals having BMI ≥ 30 (p < 0.01) (Table 2). Moreover, BMI was inversely associated with lung cancer diagnosis. Individuals who had normal weight or were underweight had the highest frequency of lung cancer diagnosis (4.9%), followed by those who were overweight (3.8%), and then obese (2.8%). Further analysis of the relationship between BMI and lung cancer diagnosis by race showed the inverse relationship still stayed for NHW and Others. However, BMI and lung cancer diagnosis among African-Americans was marginally associated (p = 0.08). BMI was also significantly associated with lung cancer histology; the frequency of adenocarcinoma decreased with obesity, while small cell lung cancer and carcinoid tumors increased slightly with obesity.

**Table 2:** Relationship of race, BMI, and lung cancer development

	**Underweight/** **normal**		**Overweight**		**Obese**		***p*-value**
	**n**	**%**	**n**	**%**	**n**	**%**	
**Race**							.001
Non-Hispanic Whites (NHW)	13,725	28.8%	20,484	43.1%	13,385	28.1%	
African-Americans	621	26.7%	917	39.3%	787	33.9%	
Others	896	30.6%	1,264	43.1%	771	26.3%	
**Lung cancer diagnosis** (*n* = 2037)							
Total	747	4.9%	867	3.8%	423	2.8%	.001
NHW	678	4.9%	794	3.8%	387	2.8%	.001
African-Americans	32	5.1%	46	4.9%	24	3.0%	.083
Others	21	4.9%	13	2.7%	2	1.2%	.041^a^
**Lung cancer Stage**							.144
Stage I & II	343	46.4%	435	50.7%	191	46.0%	
Stage III & IV	396	53.5%	423	49.3%	224	54.0%	
**Lung cancer histology type**							.03
Adenocarcinoma	331	45.0%	393	45.8%	166	39.7%	
Squamous cell carcinoma	152	20.7%	217	25.3%	89	21.3%	
Small cell carcinoma	106	14.4%	107	12.5%	72	17.2%	
Carcinoid/Neuroendocrine tumor	18	2.5%	22	2.6%	19	4.6%	
Large cell carcinoma	20	2.7%	19	2.2%	13	3.1%	
Non-Small cell carcinoma or other	109	14.8%	101	11.8%	59	14.1%	

^a^ Fisher exact test

On multivariate regression analyses (Table 3), race and BMI were associated with lung cancer diagnosis. Individuals from racial groups categorized as “Others” had lower odds of lung cancer diagnosis than Whites (aOR = 0.77, 95% CI = 0.63–0.96). In the subgroup analysis by race, BMI was inversely related to lung cancer diagnosis among Whites: those who were overweight (aOR = 0.83, 95%CI = 0.75–0.93), or obese (aOR = 0.64, 95%CI = 0.56–0.73), were less likely to develop lung cancer, compared to those with normal weight. However, this relationship was not found among African-Americans individuals who were overweight (aOR = 1.03, 95%CI = 0.64–1.66) or obese (aOR = 0.72, 95%CI = 0.41–1.25).

**Table 3:** Multivariate analysis of lung cancer diagnosis by race

	**Total**		**Whites**		**African-Americans**		**Others**	
**n**	51,930		46,554		2,299		3,077	
	**aOR**	**95% CI**	**aOR**	**95% CI**	**aOR**	**95% CI**	**aOR**	**95% CI**
**Race**								
Whites	1.00							
African-American	1.20	0.97–1.48						
Others	0.77	0.63–0.96*						
**BMI**								
Underweight/Normal	1.00		1.00		1.00		1.00	
Overweight	0.83	0.75–0.93*	0.83	0.75–0.93*	1.03	0.64–1.66	0.72	0.45–1.15
Obese	0.63	0.56–0.72*	0.64	0.56–0.73*	0.72	0.41–1.25	0.53	0.29–0.87*

Note. Adjusted for age, gender, education, family history of lung cancer, COPD, smoking status, and pack-years

*p<0.05

## Discussion

This is one of the first studies to use a large dataset to examine the racial differences in BMI and lung cancer development. Despite the small sample of African-American participants included in the NLST, this group had a significantly greater proportion of obese or morbidly obese participants compared to Whites and, therefore could be analyzed to determine the presence of an obesity paradox in lung cancer diagnosis. There was an overall inverse relationship between BMI and lung cancer development even after controlling for potential confounders. These findings were consistent with the data in lung cancer development in the previously mentioned studies [9–16]. However, this inverse relationship between BMI and lung cancer development was not significant in the African-American population. This lack of significance compared to other races could be potentially due to varying phenotypes and body composition or a small African-American cohort.

With regard to lung cancer histology type and obesity, it was found that adenocarcinoma and squamous cell carcinoma frequency decreased with increasing BMI. On the other hand, small cell lung cancer and carcinoid, both lung neuroendocrine tumors, increased with higher BMI. Obesity has been observed to be a risk factor for gastrointestinal neuroendocrine tumors [24], but there is no literature on its impact on lung neuroendocrine tumors. On the other hand, a meta-analysis found that adenocarcinoma and squamous cell carcinoma was inversely related to obesity, consistent with the results seen in this NLST analysis [13].

Even though the significance of the obesity paradox in African-Americans differs from the relationship found in the pooled analysis [21], the current study has certain limitations. First, in terms of participants, the NLST cohort was limited to subjects at high risk of lung cancer based on smoking history. Hence, cohort criteria in the NLST may not parallel the exact criteria for other screening trials and may therefore limit generalizability of results. However, lung cancer screening studies still nevertheless focus on individuals at high risk for lung cancer based on relevant criteria. Additionally, the majority of NLST participants were White and had a high education level. Further, African-Americans represented about 12.4% of the total U.S. population in 2020 [25] but the NLST only had 5% African-American participants. These study population limitations suggest that the NLST has limited generalizability in low lung cancer risk, non-smoking, lower education level, or African-American populations. Furthermore, variables like smoking could contribute as a confounding variable, given that smoking is associated with low BMI. Therefore, the obesity paradox in lung cancer risk could root back to smoking history, which is related to low BMI. Second, the NLST’s measurement of BMI was through self-reporting. Therefore, participant BMI may have been over- or under-reported and could contribute to random error in statistical analysis of the obesity paradox as it may not be indicative of the patients’ true BMI. Third, BMI measurement does not account for differences in individual body composition, with individually varying lean body mass, subcutaneous fat, and visceral fat. Specifically, African-American individuals have high lean body mass and subcutaneous fat but low visceral fat, despite having a generally higher BMI, while White individuals have been reported to have relatively higher visceral fat [26, 27]. The generally lower visceral fat among African-Americans is a possible factor that may contribute to differences in association between BMI and lung cancer diagnosis. This hypothesis should be explored further. Additionally, body distribution, specifically a greater waist circumference (WC) and waist to hip ratio (WHR), has been found to have a statistically significant positive association with lung cancer risk in African-Americans and Whites [21, 28, 29]. However, these phenotypic measures are not reflected in BMI and should be explored further as potential risk factors for lung cancer development, given the fact that African-Americans are disproportionately affected by lung cancer [30–32].

Nevertheless, this study has its strengths. It was a large-scale screening study, which allows for closer analysis of high-risk individuals. Being able to detect risk factors or protective factors earlier would allow for proactive screening among vulnerable lung cancer populations. Additionally, it is one of few studies to examine racial and ethnic differences in obesity and lung cancer diagnosis in a large cohort.

In conclusion, this study found that there was no significant relationship between BMI and lung cancer diagnosis among African-American individuals undergoing lung cancer screening. This study’s findings differ from the results of the previously described and limited literature on race and obesity in lung cancer diagnosis. Future research should focus on body composition and distribution and its relationship with lung cancer diagnosis in NLST screening data to improve screening efforts and catch high-risk patients.

## Data Availability

The data that support the findings of this study are available from National Cancer Institute’s Cancer Data Access System but restrictions apply to the availability of these data, which were used under license for the current study, and so are not publicly available. Data are however available from the authors upon reasonable request and with permission of NCI.
